# Intraocular Pressure Spikes following Neodymium-doped Yttrium Aluminum Garnet Laser Capsulotomy: Current Prevalence and Management in Israel

**DOI:** 10.5005/jp-journals-10028-1225

**Published:** 2017-08-05

**Authors:** Asaf Achiron

**Affiliations:** Resident, Department of Ophthalmology, Edith Wolfson Medical Center Holon, Israel

**Keywords:** Apraclonidine, Cataract surgery, Intraocular pressure, Neodymium-doped yttrium aluminum garnet laser capsulotomy, Posterior capsular opacification.

## Abstract

**Aim:**

The current treatment for posterior capsular opacification (PCO), neodymium-doped yttrium aluminum garnet (Nd:YAG) laser capsulotomy, may lead to increased intraocular pressure (IOP). Our aim was to survey routines in the management of IOP spikes and to identify the rate of IOP spikes following prophylactic apraclonidine treatment.

**Materials and methods:**

A survey questionnaire among ophthalmologists and a retrospective registry review was used. Patients were administered apraclonidine 0.5% prior to capsulotomy. The IOP was measured before and 1 hour postprocedure.

**Results:**

A total of 71% of responders (n = 45) routinely prescribe topical IOP-lowering medication and 82% routinely measure IOP before or after capsulotomy. The registry analysis included 87 eyes of 75 patients. Mean IOP decreased by 0.9 ± 3.3 mm Hg (p = 0.01, range: -6 to 10) following capsulotomy. No patient reached IOP values above 21 mm Hg following the procedure, with 3.4 and 1.1% of patients demonstrating an IOP elevation of more than 3 and 5 mm Hg respectively. No association was found between number of laser shots, mean laser power, or comorbid conditions, such as diabetes, hypertension, or glaucoma status with posttreatment IOP.

**Conclusion:**

Most ophthalmologists surveyed routinely prescribe prophylactic IOP-lowering medication and measure IOP before or after capsulotomy. Mean IOP remained clinically stable following capsulotomy with prophylactic apraclonidine instillation, and no patient reached IOP values above 21 mm Hg. Differences in laser delivery or comorbid conditions were not associated with posttreatment IOP. Considering that no patient demonstrated a clinically significant IOP spike following prophylactic apraclonidine instillation, perhaps routine measurement of IOP following primary Nd:YAG laser may be reserved for high-risk patients only.

**Clinical significance:**

In this work, we showed the prophylactic effect of apraclonidine 0.5% and suggest that measuring IOP after the procedure is necessary only in certain high-risk cases, possibly helping to reduce workload and patient waiting time and improving quality of service.

**How to cite this article:**

Achiron A. Intraocular Pressure Spikes following Neodymium-doped Yttrium Aluminum Garnet Laser Capsulotomy: Current Prevalence and Management in Israel. J Curr Glaucoma Pract 2017;11(2):63-66.

## INTRODUCTION

Posterior capsular opacification is the most common postoperative complication of cataract surgery with a cumulative 5-year incidence of H.9%.^1^ The treatment of choice for PCO is short pulsed NdYAG laser applied to the posterior capsule in order to create an opening in the visual axis. Although considered safe, it may result in retinal detachment, lens subluxation, and lens pitting. The most common complication is a transient increase in IOP, which may occur in 15 to 36% of patients who receive no prophylactic treatment.^2-6^ This IOP spike may lead to additional injury in eyes with advanced glaucomatous optic nerve damage.^2^

Several controlled studies from recent decades have investigated IOP-lowering agents and demonstrated successful control of IOP.^3-12^ Routine practice and outcomes in real-life settings, however, have not been as thoroughly explored. Apraclonidine 0.5% is a common choice as a prophylactic treatment prior to laser capsulotomy.^8,13^ Reported rates of IOP spikes above 5 mm Hg following prophylactic apraclonidine 0.5% and capsulotomy vary between 2 and 8.5%.^2,5,11,12^

The purpose of this study was to survey routines in the management of post Nd:YAG laser capsulotomy IOP spike in Israel and to identify the rate of postprimary NdYAG laser capsulotomy IOP spikes following one drop of prophylactic apraclonidine 0.5% treatment.

## MATERIALS AND METHODS

### Survey Questionnaire

Questionnaires were sent via e-mail to ophthalmologists registered at the Tel Aviv University Continuous Medical Education (CME) network. Ophthalmologists were asked to complete a 14-question internet-based survey regarding their IOP management routine, following cap-sulotomy (https://goo.gl/forms/87ZCQMO0l89sFqow2).

### Retrospective Registry Review

Charts of patients who had undergone primary Nd:YAG laser capsulotomy were reviewed. Demographic data, time since cataract surgery, and relevant medical diagnoses were extracted. Patients who had previously undergone Nd:YAG laser capsulotomy in the same eye were excluded. All subjects were treated with one drop of apraclonidine 0.5% prior to the procedure and another physician measured IOP 1 hour posttreatment using Goldmann applanation tonometry. Change in IOP was calculated for each patient as the difference between pre-and posttreatment IOPs.

### Statistical Analysis

Descriptive data and the proportion of patients who had IOP > 21 mm Hg were calculated using Statistical Package for the Social Sciences version 17 By IBM Inc. Correlation analysis between AIOP (IOP before and after treatment) and visual acuity was conducted in addition to three logistic regression analyses. Successful outcomes following treatment were defined as those having an IOP < 21 mm Hg, absolute change in IOP ≤3 and ≤5 mm Hg, and IOP change <20%. This study was approved by the Edith Wolfson Institutional Review Board Committee.

## RESULTS

### Online Survey

Total response rate for the survey questionnaire was 54% (45 of 83), responses were received from two-thirds of all certified ophthalmology teaching departments in Israel (13/19). The mean age of responders was 36.6 ± 8.4 years, 65.9% were male, 75.0% were residents, and the median of experience in ophthalmology practice was 3.0 years. The IOP measurement was routinely performed before and/or after the procedure by 81.8% of responders (68.2% only before, 68.2% only after); 18.2% do not measure IOP at all and 54.5% responded that they measured IOP twice (before and after capsulotomy).

Topical medication to lower IOP before and/or after the capsulotomy was prescribed by 74.4% of responders (52.3% only before, 38.1% only after); 25.6% do not initiate any prophylactic IOP reduction treatment and 16.3% responded that they routinely prescribe topical medication twice (before and after capsulotomy).

Of responders who routinely prescribe topical IOP-lowering medication, 81.8% also measure, at least once, the IOP (54.5% before and 59.1% after capsulotomy). Only 54.5% of responders who do not prescribe any topical antiglaucoma therapy also measure IOP at least once. Average time from preventive IOP treatment to capsu-lotomy was 15.9 ± 14.6 minutes (median 7.5 minutes).

### Retrospective Registry Review

A total of 87 eyes of 75 patients (62.1% female) were included in the study. One eye of one patient was excluded because the patient had undergone previous Nd:YAG laser capsulotomy in that eye. The mean age was 75.6 ± 9.3 years; 29.9% had diabetes, 64.4% had systemic hypertension, and 14.9% had glaucoma (n = 13, all were treated). Mean time from cataract surgery was 32.0 ± 22.4 months. The mean number of laser shots was 21.8 ± 12.2 and mean laser power was 3.1 ± 0.5 mJ.

Following capsulotomy and apraclonidine prophylactic treatment, average IOP remained clinically stable (14.0 ± 2.6 to 13.1 ± 2.9 mm Hg, IOP change: -0.9 ± 3.3 mm Hg, range: -6 to 10; p = 0.01). Three cases (3.4%) experienced an increase in IOP of more than 3 mm Hg and only one case (1.1%) more than 5 mm Hg.

**Graph 1:**
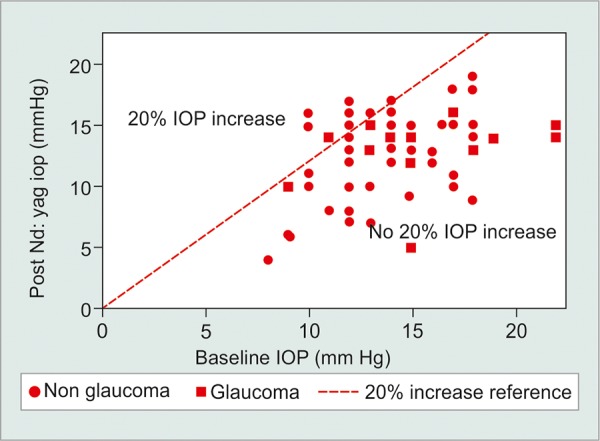
Scatter plot of post Nd:YAG laser capsulotomy IOP *vs* baseline IOP. Squares represent eyes of patients with glaucoma and circles represent eyes of patients without glaucoma. The dashed reference line represents an increase of 20% or more from baseline IOP. None of the eyes demonstrated a clinically significant rise in IOP over 21 mm Hg

Subgroup analysis revealed that glaucoma patients had a higher IOP prior to treatment compared with nonglaucoma patients (15.6 ± 3.9 *vs* 13.6 ± 2.2 mm Hg respectively, p = 0.01). There was no significant posttreatment difference in the mean IOP (13.1 *vs* 13.0 mm Hg, p = 0.9). The absolute change in IOP, however, was larger in the glaucoma patients group (ΔIOP: -2.6 ± 4.0 *vs* -0.57 ± 3.0 mm Hg, p = 0.03). None of the eyes demonstrated a clinically significant rise in IOP (Graph 1).

No association was found between number of laser shots, mean laser power, or absolute amount of laser delivered (number of shots multiplied by the power) to either posttreatment IOP or IOP change.

## DISCUSSION

Acute IOP elevation following capsulotomy is common among patients who do not receive prophylactic treatment and may occur in 15 to 36% of cases.^2,4-7,9^ When prophylactic treatment is prescribed, however, an IOP spike above 5 mm Hg is seen in only 2 to 8.5% of cases.^2^ The increased IOP may be caused by reduced aqueous humor outflow facility due to capsular debris, cells (erythrocytes, lymphocytes, and macrophages), fibrin, pigment, and other materials that may clog the trabecular meshwork. Shock waves created by the laser may also damage the trabecular meshwork, causing clogs.

In our study, patients received prophylactic apra-clonidine and 1.1% of cases (1 of 87) experienced an IOP increase above five, with no patients reaching IOP values above 21 mm Hg. These results are in line with those of other researchers when using prophylactic IOP-lowering medication. Holweger and Marefat^8^ reported that 1% of 101 patients had IOP spikes over 5 mm Hg following their capsulotomies. Barnes et al^2^ reported a 2% rate among 47 patients treated with either acetazolamide or apracloni-dine. Unal et al^11^ examined 115 patients and reported a stable IOP following apraclonidine and capsulotomy (from 16.1 to 15 mm Hg) and a relatively high rate of IOP spikes > 5 mm Hg seen in 8.6% (5/58) of their patients.

Apraclonidine 0.5% was effective in our glaucoma subgroup, with a mean IOP reduction of 2.6 ± 4.0 mm Hg (95% confidence interval: -2.0 to -0.95). Moreover, glaucoma status was not a contributor for the risk of an IOP spike in our cohort. Barnes et al^2^ also reached a similar conclusion, showing that only 4% (1/23) of glaucoma patients who received apraclonidine prior to their capsu-lotomies experienced an IOP rise greater than 5 mm Hg.

We did not find any of the investigated laser parameters to have a significant clinical effect on IOP change or posttreatment values. This is in contrast to a report by Ari et al,^14^ who demonstrated that higher laser power is associated with a greater IOP spike.

One possible implication arising from these results is the possibility to forego the 1 hour postlaser IOP measurement. Long waiting times are a major source of patient dissatisfaction and may adversely affect clinical outcomes and patient compliance with treatment regimens; waiting times have been found to be an important factor in rating service quality and overall patient satisfaction.^15^ It is therefore important to critically assess every decision affecting patient waiting times in the clinic. Given the low rates of IOP spikes in patients treated with IOP-lowering medication, reported here and in other studies, it might be reasonable to consider this avenue of action in low-risk patients.

Our survey included responses on the management of IOP spike following capsulotomy from two-thirds of all certified teaching departments in Israel (13/19). By targeting the university CME network contacts list, we had achieved high response rate from residents. As residents perform capsulotomy from an early stage in their residency, the survey results encompassed current practice and were exposed to limited recall biases.

To conclude, most ophthalmologists surveyed routinely prescribe prophylactic IOP-lowering medication and measure IOP before or after capsulotomy. Mean IOP remained clinically stable following capsulotomy with prophylactic apraclonidine instillation. Increase in IOP above 5 mm Hg was observed in 1.1% of cases (1/87) and no patients reached IOP values above 21 mm Hg following the procedure. Differences in laser delivery or comorbid conditions were not associated with posttreatment IOP.

## CLINICAL SIGNIFICANCE

In this work, we showed the prophylactic effect of apra-clonidine 0.5% and suggest that measuring IOP after the procedure is necessary only in certain high-risk cases, possibly helping to reduce workload and patient waiting time and improving quality of service.
